# Unveiling the Pre‐Weaning Growth Performance and Some Reproductive Characteristics of Akkaraman and Central Anatolian Merino Sheep

**DOI:** 10.1002/vms3.70221

**Published:** 2025-02-24

**Authors:** Sedat Behrem

**Affiliations:** ^1^ Faculty of Veterinary Medicine, Department of Animal Science Aksaray University Aksaray Türkiye

**Keywords:** Akkaraman, Central Anatolian Merino, environmental factors, lamb livability, reproductive traits

## Abstract

This study evaluated growth and reproductive traits in two sheep breeds in Türkiye, Akkaraman (AKK) and Central Anatolian Merino (CAM), with a focus on the impact of non‐genetic factors. Data were analysed from 21,414 AKK and 20,099 CAM lambs for birth weight (BW) and 27,528 AKK and 24,639 CAM lambs for weaning weight (WW), average daily weight gain (ADWG) and Kleiber ratio (KR) using a linear mixed model. AKK lambs showed a significantly higher mean BW (4.19 ± 0.01 kg) than CAM lambs (4.02 ± 0.01 kg) (*p* < 0.001), whereas CAM lambs had significantly higher WW, ADWG and KR (*p* < 0.001 for each trait). Fixed effects of sex, birth type, dam age, birth year, herd size and season significantly influenced all growth traits (*p* < 0.05). Male lambs demonstrated higher BW, WW, ADWG and KR than females (*p* < 0.001), and single‐born lambs had significantly higher BW and WW than twins (*p* < 0.001). Dam age also influenced all growth traits, with lambs from younger dams tending to be heavier (*p* < 0.001). In terms of survival, AKK lambs exhibited a significantly higher survival rate (93.85%) than CAM lambs (89.50%) (*p* < 0.001). Conception rates were similar for both breeds (92.9% for AKK and 92.3% for CAM), whereas CAM lambs showed higher fecundity and litter size. These findings underscore the breed differences in growth and reproductive traits and highlight the importance of considering non‐genetic factors to inform breed‐specific management practices aimed at optimizing productivity.

## Graphical abstract

This study evaluates growth and reproductive traits in Akkaraman (AKK) and Central Anatolian Merino (CAM) sheep in Türkiye. AKK lambs showed higher birth weights but lower weaning weights and growth rates compared to CAM lambs. Significant non‐genetic factors, such as sex, birth type and dam age, influenced these traits. AKK lambs exhibited higher survival rates, while CAM demonstrated greater fecundity. These findings underscore the importance of breed‐specific management practices to optimize productivity.

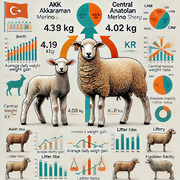

## 1 Introduction

Sheep have been among the most widely utilized animals since their domestication, owing to their resilient and adaptable nature. This adaptability has enabled sheep to thrive under diverse and often challenging climatic and environmental conditions, facilitating their widespread production worldwide. Consequently, sheep have become a critical source of red meat, contributing significantly to global food production (Talebi et al. 2022). Moreover, when compared to the production system of large animals, sheep farming offers a range of advantages that have promoted the widespread adoption of the diversification in breeding activities (Joy et al. 2020; Kizilaslan et al. 2022). Türkiye holds a significant share globally with 56 million small ruminants (79.4% sheep, 20.6% goats) (TUIK 2023). The unique geography of Türkiye, with its diverse climatic conditions, has fostered the development of numerous highly adapted sheep breeds over the centuries (Yalcin 1986). In Türkiye, native sheep breeds account for 91.14%, whereas Merino crossbreeds make up 8.86% of the sheep population; these native and crossbred breeds have adapted to different regions of Anatolia (TUIK 2023). Sheep farming accounts for approximately 20% (TUIK 2022) of total red meat production and provides advantages to the rural economy of Türkiye, making significant contributions to its development and sustainable production in Türkiye.

Türkiye has great richness with more than 20 native sheep breeds and many crossbreeds that have been obtained from crossbreeding between exotic and native sheep (FAO 2023; Ertan et al. 2025). Among its native breeds, the Akkaraman (AKK) sheep, known for its fat‐tailed, meat production and long wool characteristics, stands out as the most widely raised breed (Arzik et al. 2023, 2024). Moreover, AKK sheep have played a role in creating the Central Anatolian Merino (CAM) (comprising 85% German Meat Merino and 15% AKK) by combining efforts for the purposes of improving both meat and wool production. CAM has become preferred among farmers due to its adaptability to poor pastures, its resistance to diseases and its ability to the harsh environmental conditions (Arzik et al. 2022; Kizilaslan et al. 2023; Yalcin 1986). Although many studies have shown unsuccessful attempts at crossbreeding in different regions of the world (Kosgey et al. 2006), crossing studies on CAM sheep can provide a good example for breeding programmes. The absence of regular recording and the reliance on phenotypic selection are recognized as common issues among small ruminant breeders in pasture‐based production systems in Türkiye (Ceyhan et al. 2019). However, to address these problems and to meet the growing demand for meat, the ‘National Sheep and Goat Breeding Project’ was initiated in 2005 by the Minister of Agriculture and Forestry.

The systematic selection approach for sheep breeding begins with characterizing and improving the impact of non‐genetic factors and is subsequently followed by a genetic selection to achieve cumulative improvements (Arzik et al. 2022; Sönmez et al. 2009). In addition to the selection criteria regarding growth performance, reproductive performance plays a critical role in the field of animal husbandry, exerting a significant impact on the economic success and sustainability of the enterprise (Unlusoy 2023). In the selection program for sheep production, the economically important traits are lamb birth weight, weaning weight (WW), feed efficiency and average daily gain as well as reproductive traits, such as conception rate, fecundity, litter size and fecundity at weaning. In addition to these economic traits, the Kleiber ratio (KR) is another important indicator of growth rate and is traditionally employed for the assessment of feed efficiency (Mahala et al. 2020; Supakorn and Pralomkarn 2012). Moreover, genetic improvement programmes that incorporate molecular techniques and marker‐assisted selection (MAS) are essential for the efficient introgression of major genes, providing valuable insights into gene regulation and expression and enabling the development of superior livestock breeds with enhanced reproductive and growth traits (Majd et al. 2019; Talebi et al. 2018, 2022, 2023, 2024).

The AKK sheep breed is notable for being a highly adaptable native breed, whereas the CAM breed is an efficient crossbreed resulting from the crossbreeding of AKK and German Meat Merino and excels in meat production. Therefore, the aim of this study was to assess the specific growth and reproductive traits, as well as the effect of non‐genetic factors on these traits, and to compare these traits between CAM and AKK sheep in Türkiye.

## 2 Materials and Methods

### 2.1 Geographic Circumstances and Animal Management

The Ankara province is in the northwest of the Central Anatolian Region of Türkiye, at 39° 55′ north latitude and 32° 50′ east longitude. Although the region's pastures, totalling 411,000 ha in Ankara, are partially characterized by mixed steppe meadows containing grasses, legumes and other plant families, they are predominantly made up of low‐quality and low‐nutrient plants (Ünal et al. 2012). During the summer months, the vegetation nearly dries out. In the summer period, animals usually graze on agricultural lands, mainly on harvest residues.

Feeding practices are consistent across farms for both AKK and CAM breeds. The animals graze in the natural pasture between April and November. Lambing typically occurs in the winter and spring (December to April) periods when the ewes are fed in barns. From December to April, lambs are kept with their dam, and the ewes are fed with roughage (i.e., alfalfa, wheat straw and vetch) along with an average of 1.5 kg/day of concentrated feed (with an average energy content of 2600 kcal/kg, the feed contains 15% crude protein, 2% crude fat, 10% crude fibre and 9% crude ash) per animal. The lambs are supplemented with alfalfa‐based concentrate feed as a starter diet. In spring, feeding practices are adjusted on the basis of the climate, continuing with maternal milk and allowing lambs to graze on pastures.

Most births occur during winter (December, January and February) and extend into early spring (March and April). Before the lambing period, the breeders were equipped with ear tags and digital handheld scales for recording purposes as part of the project. Within 24 h of birth, each lamb's details—number, birth weight, birth date, type, sex and maternal ear tag number—were recorded during weighing. At the end of the birthing season, the records were collected along with the recording devices. A specific date was scheduled for weighing the lambs at weaning, and their weights were measured and recorded on the farm.

### 2.2 Animal Material and Phenotypes

The animals used in the study were obtained from two subprojects, coded TAGEM/06OAM2012‐01 and TAGEM/06AKK2011‐01. These subprojects were conducted under the National Small Ruminant Breeding Project, which is a collaborative effort involving universities, research institutes and sheep and goat breeders’ associations. The 30 different AKK and 29 different CAM herds used in the study were registered to the Ankara sheep and goat breeders’ association, which kept regular records about these flocks. This study involved the collection of data spanning from 2014 to 2017 for two breeds: AKK and CAM lambs. The dataset comprised 21,414 records for birth weight (BW) and 27,528 records for WW in the case of the AKK lambs and 20,099 records for BW and 24,639 records for WW for the CAM lambs. Additionally, information on the average daily weight gain (ADWG) and the KR was also gathered.

#### 2.2.1 Growth Traits

The regular records encompassed details such as birth and weaning dates, the dams’ age (categorized as 2–7 years and above), herd size (categorized as <150, 150–300 and >300), sex, birth type (singlets/twins) and lambing season (winter/spring). BW was recorded within the first 24 h following lambing. WW is the adjusted 90 days of weight of the lambs with linear interpolation of weaning records. a weight. ADWG was calculated using linear statistics based on BW and WW. Furthermore, the KR was derived from the ADWG and WW values (ADWG/WW^0.75^). The data structure and sample size, detailed in Table 1, were thoroughly explained after the removal of outliers.

**TABLE 1:** Descriptive statistics of pre‐weaning growth traits.

Trait	BW (kg)		WW (kg)		ADWG (g)		KR	
Breed	AKK	CAM	AKK	CAM	AKK	CAM	AKK	CAM
**Number of observations**	21,414	27,528	20,099	24,639	20,099	24,639	20,099	24,639
**Mean**	4.28	4.07	25.94	26.76	240.70	252.13	20.52	21.05
**Standard error**	0.01	0.01	0.05	0.05	0.58	0.52	0.02	0.02
**Minimum**	1.10	1.43	8.33	8.23	23.95	29.02	4.64	5.65
**Maximum**	8.99	8.39	51.03	51.96	516.94	543.08	27.88	28.23
**Coefficient of variation**	23.44	20.39	28.91	27.36	34.06	32.31	14.26	13.23

Abbreviations: ADWG, average daily weight gain; AKK, Akkaraman; BW, birth weight; CAM, Central Anatolian Merino; KR, Kleiber ratio; WW, weaning weight.

#### 2.2.2 Reproductive Traits

Records on AKK and CAM farms are consistently observed throughout the ewes’ pregnancy period. Specific reproductive performance traits, such as conception rate, fecundity, litter size, fecundity at weaning, litter size at weaning and the survival rate in the sheep, were obtained from these records.

### 2.3 Statistical Analyses

#### 2.3.1 Growth Traits

The linear mixed models were used to assess the impact of various factors and to estimate the least square means (LSMs) for the traits. Additionally, diagnostic tests were carried out to evaluate the models and examine the interaction among the factors. To formulate our ultimate linear mixed models, we initially investigated the influence of environmental factors on growth and survival and reproductive parameters, including but not limited to breed, sex, birth type, mother age, birth year, herd size and birth season. For data management and statistical analysis, we harnessed the power of critical R packages such as ‘lme4’, ‘lmerTest’ and various other foundational tools within the R statistical environment (R Core Team 2023).

Following the fitting of the final models for various traits, we used the identified linear mixed models to assess the influence of non‐genetic factors on the growth and reproductive traits. These models allowed us to establish the LSMs for the considered factors. To discern the differences between the significant factor groups, Duncan's multiple range test (DMRT) was employed. Detailed descriptions of the final linear mixed models for each trait are provided in the following equation:

$$\begin{array}{ccc} \mathrm{Model}\;\;:\;y_{\mathrm{ijklnopu}} & = & \mu +g_{i}+{\mathrm{sex}}_{j}+bt_{k}+m_{l}+{\mathrm{year}}_{n}\\ & & +\;{\mathrm{herd}}_{o}+{\mathrm{season}}_{p}+e_{\mathrm{ijklnopu}} \end{array}$$

where *y*_ijklnopu_ is the response variable of BW, WW, ADWG and KR; *μ* is the intercept; g_i_ is the fixed effect of the breed (2 levels); sex_*J*_ is the fixed effect of sex (two levels); *bt*_*k*_ is the fixed effect of birth type (two levels: singlet and multiples); *m*_*l*_ is the fixed effect of dam age (six levels: categorized as 2–7 years and above); year_*n*_ is the fixed effect of birth year (four levels: 2014–2017); herd_*o*_ is the fixed effect of herd size (three levels: categorized as <150, 150–300 and >300); season_*p*_ is the fixed effect of birth season (two levels); Finally, the term *e*_ijklnopu_ is the residual error for individual measurement, accounting for the variability in observations within the models.

#### 2.3.2 Reproductive Traits

The calculation for reproductive parameters was implemented on the basis of the record of ewes during mating and lambing time. The calculation methods for these parameters are detailed in the following equation:

(1) $$F\;\left(\%\right)=\frac{\mathrm{EL}}{\mathrm{NEER}}\;\times \;100$$

(2) $$\mathrm{FE}\;\left(\%\right)=\frac{\mathrm{NLB}}{\mathrm{NEER}}\;\times \;100$$

(3) $$\mathrm{LS}=\frac{\mathrm{NLB}}{\mathrm{EL}}$$

(4) $$\mathrm{LSW}=\frac{\mathrm{NLW}}{\mathrm{EL}}$$

(5) $$\mathrm{FEW}=\frac{\mathrm{NLW}}{\mathrm{NEER}}$$

(6) $$\mathrm{SR}\;\left(\%\right)=\frac{\mathrm{NLW}}{\mathrm{NLB}}\;\times \;100$$

where EL is the number of ewes lambing; *F* is conception rate; NEER is the number of ewes exposed to the ram; FE is fecundity; NLB is the number of lambs born; LS is litter size; FEW is fecundity at weaning; NLW is the number of lambs weighted on Day 90; LSW is a litter size at weaning; SR is survival rate (%).

To analyse the influence of various non‐genetic factors—such as breed, sex, birth type, birth year, dam age, herd size and season—on the observed lamb survival data, a Chi‐square test was employed. The analysis was conducted using a sample dataset representative of the AKK and CAM populations, ensuring that all relevant factors were accounted for.

## 3 Results

### 3.1 Growth Traits

The descriptive statistics of the data, including the sample size, for the traits BW, WW, ADWG and KR related to AKK and CAM lambs are presented in Table 1. The effect of environmental factors, including sex, birth type, dam age, birth year, herd size and season, on the BW, WW, ADWG and KR traits in the AKK and CAM lambs is estimated and illustrated in Table 2.

**TABLE 2:** The least square means (± standard error [SE]) of the pre‐weaning traits in Akkaraman and Central Anatolian Merino lambs.

	BW (kg)			WW (kg)			ADWG (g)			KR		
Fixed effects	*n*	LSM ± SE	*p* value	*n*	LSM ± SE	*p* value	*n*	LSM ± SE	*p* value	*n*	LSM ± SE	*p* value
**Breed**			***			***			***			***
AKK	21,414	4.19 ± 0.01^a^		20,099	26.69 ± 0.07^b^		20,099	249.99 ± 0.72^b^		20,099	20.85 ± 0.03^b^	
CAM	27,528	4.02 ± 0.01^b^		24,639	27.45 ± 0.06^a^		24,639	260.32 ± 0.63^a^		24,639	21.33 ± 0.02^a^	
**Sex**			***			***			***			***
Male	24,123	4.14 ± 0.01^a^		22,122	27.65 ± 0.06^a^		22,122	261.28 ± 0.67^a^		22,122	21.28 ± 0.02^a^	
Female	24,819	4.08 ± 0.01^b^		22,616	26.49 ± 0.06^b^		22,616	249.03 ± 0.67^b^		22,616	20.89 ± 0.02^b^	
**Birth type**			***			***			NS			*
Single	34,086	4.21 ± 0.01^a^		32,539	27.43 ± 0.05^a^		32,539	257.99 ± 0.53^a^		32,539	21.11 ± 0.02^a^	
Twin	14,856	4.00 ± 0.01^b^		12,199	26.71 ± 0.08^a^		12,199	252.32 ± 0.84^a^		12,199	21.06 ± 0.03^b^	
**Dam age (year)**			***			***			***			***
2	8909	4.17 ± 0.01^a^		8411	27.03 ± 0.09^a^		8411	254.03 ± 0.97^ab^		8411	20.99 ± 0.03^b^	
3	8647	4.12 ± 0.01^bc^		7800	27.47 ± 0.09^a^		7800	259.45 ± 1.01^a^		7800	21.21 ± 0.04^a^	
4	10,099	4.14 ± 0.01^b^		9573	26.61 ± 0.08^c^		9573	249.70 ± 0.91^c^		9573	20.93 ± 0.03^b^	
5	6954	4.08 ± 0.01^d^		6262	26.93 ± 0.09^b^		6262	253.81 ± 1.08^bc^		6262	21.06 ± 0.04^b^	
6	7498	4.00 ± 0.01^e^		6611	27.32 ± 0.09^ab^		6611	259.05 ± 1.05^a^		6611	21.25 ± 0.04^a^	
7 and upon	6835	4.14 ± 0.01^c^		6081	27.08 ± 0.09^ab^		6081	254.89 ± 1.08^ab^		6081	21.05 ± 0.04^b^	
**Birth year**			***			***			***			***
2014	12,089	4.14 ± 0.01^a^		11,000	25.93 ± 0.08^d^		11,000	242.13 ± 0.89^d^		11,000	20.77 ± 0.03^d^	
2015	12,124	4.16 ± 0.01^a^		11,118	29.09 ± 0.08^a^		11,118	277.01 ± 0.85^a^		11,118	21.49 ± 0.03^a^	
2016	13,107	4.04 ± 0.01^c^		11,692	26.30 ± 0.07^c^		11,692	247.39 ± 0.82^c^		11,692	20.86 ± 0.03^c^	
2017	11,622	4.10 ± 0.01^b^		10,928	26.97 ± 0.08^b^		10,928	254.10 ± 0.87^b^		10,928	21.21 ± 0.03^b^	
**Herd size**			***			***			***			***
<150	10,029	3.97 ± 0.01^b^		9455	26.74 ± 0.08^c^		9455	253.04 ± 0.91^b^		9455	21.08 ± 0.03^b^	
150–300	14,861	4.15 ± 0.01^a^		13,129	26.82 ± 0.08^b^		13,129	251.91 ± 0.84^c^		13,129	20.97 ± 0.03^c^	
>300	24,052	4.21 ± 0.01^a^		22,154	27.65 ± 0.06^a^		22,154	260.52 ± 0.63^a^		22,154	21.20 ± 0.02^a^	
**Season**			***			***			***			***
Winter	37,698	4.08 ± 0.01^b^		35,906	25.43 ± 0.04^b^		35,906	237.26 ± 0.49^b^		35,906	20.55 ± 0.02^b^	
Spring	11,232	4.14 ± 0.01^a^		8832	28.72 ± 0.08^a^		8832	273.05 ± 0.91^a^		8832	21.61 ± 0.03^a^	
**Intercept**	48,942	4.04 ± 0.02		44,738	28.64 ± 0.15		44,738	273.31 ± 1.61		44,738	21.85 ± 0.06	

*Note*: The mean values which have different superscripts are significantly different. *n*, number of observations.

Abbreviations: AKK, Akkaraman; ADWG, average daily weight gain; BW, birth weight; CAM, Central Anatolian Merino; LSM, least square mean; NS, not significant; WW, weaning weight.

^***^ *p* < 0.001.

^**^ *p *< 0.01.

^*^ *p *< 0.05.

The study revealed a statistically significant (*p* < 0.001) difference between the two breeds, with the LSM values for BW estimating 4.19 ± 0.01 kg for AKK and 4.02 ± 0.01 kg for CAM. However, singlet lambs displayed higher LSM values for BW compared to twin lambs, and male lambs had greater than their female counterparts. Notably, lambs born to younger dams (aged 2–4 years) exhibited higher (*p* < 0.001) LSM values for BW compared to other age groups (aged 5–7 and above). As seen in Table 2, farms with a herd size of 150 or more recorded higher birth weights (*p* < 0.001). Spring‐born lambs exhibited greater birth weights compared to those from smaller scale farms (*p* < 0.001).

Regarding the WW, the AKK lambs had a mean WW of 26.69 ± 0.07 kg, whereas the CAM lambs had a mean WW of 27.45 ± 0.06 kg, and the difference between the breeds was significant in the study (*p* < 0.001). The WWs for the different categories are presented in detail in Table 2. The findings of the study highlighted that all the investigated fixed variables had a substantial influence on WW in both the AKK and CAM lambs (*p* < 0.001). Additionally, the sheep born in 2015 had notably higher WWs than those born in other years.

The result showed that the differences between the ADWG of the CAM and AKK lambs were significant (*p* < 0.001). The CAM lambs had 10.33 g more ADWG than the AKK lambs (Table 2). The effects of sex, dam age, birth year, herd size and season on the daily live weight gain of the lambs were found to be significant (*p* < 0.001), whereas the effect of birth type was found to be non‐significant.

The other feature, KR, was found to be 21.33 ± 0.02 in the CAM lambs and 20.85 ± 0.03 in the AKK lambs, and a statistically significant difference between the two breeds was observed (*p* < 0.001). It can be seen in Table 2 that the effects of sex, dam age, year, herd size and season, among the affecting non‐genetic factors, all had statistical significance (*p* < 0.001) on KR, whereas birth type did not have any effect on KR (*p* < 0.05). In the study, the KR value was 21.11 ± 0.02 in single‐born lambs, 21.25 ± 0.04 in lambs born from 6‐year‐old dams, 21.49 ± 0.03 in 2015, 21.20 ± 0.02 in lambs at large farms and in lambs born in the spring season. These findings are presented in detail in Table 2.

### 3.2 Lamb Survival Rate and Some Reproductive Performance of Ewes

Table 3 presents the survival rates of the lambs from birth to weaning along with some reproductive parameters of the ewes. The survival rates of the lambs were compared using the Chi‐square test across various environmental factors (breed, sex, birth type, birth year, dam age, herd size and season). The results indicated a statistically significant difference (*p* < 0.05) in survival rates between the two breeds, with the AKK lambs exhibiting higher survival rates (93.85%) compared to the CAM lambs (89.50%). When comparing the survival rates based on birth type, as expected, single‐born lambs had higher survival rates (95.46%) than twins (82.11%) from birth to weaning (*p* < 0.05). Similarly, a statistically significant difference in lamb survival rates was observed across different years (*p* < 0.05). However, statistical analyses revealed that the factors, such as the lambs’ gender, the age of the dams, the herd size and the birth seasons, did not significantly affect survival rates (*p* > 0.05).

**TABLE 3:** Livability of lambs at weaning (SR120) and some reproductive traits of ewes.

	Livability					Reproductive traits				
Factor	NB (*n*)	NB90 (*n*)	SR90 (%)	Chi‐sq. value	*p* value	Conception rate (%)	Fecundity	Fecundity at weaning	LS	LSW
**Breed**				24.978	0.00					
AKK	21,414	20,099	93.85			92.90	1.04	0.98	1.12	1.05
CAM	27,528	24,639	89.50			92.30	1.16	1.04	1.26	1.12
**Sex**				0.452	0.50					
Male	24,123	22,122	91.70							
Female	24,819	22,616	91.12							
**Birth type**				201.621	0.00					
Single	34,086	32,539	95.46							
Twin	14,856	12,199	82.11							
**Dam age (year)**				2.870	0.72					
2	9205	8411	91.81							
3	8618	7800	91.35							
4	10,335	9573	92.98							
5	6901	6262	91.58							
6	7213	6611	92.56							
7 and upon	6670	6081	92.06							
**Birth year**				16.038	0.00					
2014	12,089	11,000	90.99			93.80	1.05	0.96	1.13	1.03
2015	12,124	11,118	91.70			93.20	1.12	1.03	1.20	1.10
2016	13,107	11,692	89.20			88.70	1.09	0.97	1.24	1.10
2017	11,622	10,928	94.02			94.65	1.12	1.06	1.18	1.09
**Herd size**				0.065	0.96					
<150	10,344	9445	91.40							
150–300	14,388	13,129	91.24							
>300	24,210	22,154	91.50							
**Season**				0.228	0.63					
Winter	39,236	35,906	91.88							
Spring	9706	8832	92.78							

*Note*: NB, the number of live‐born lambs; NB90, the number of lambs that survived to weaning (90 days of age); SR90, the livability of lambs at weaning; LSB, litter size at birth; LSW, litter size at weaning.

Table 3 presents the reproductive performance characteristics of the AKK and CAM ewes. The study found that in the AKK lambs, the average conception rate rate was 92.9% over the years. Conversely, for the CAM lambs, the average conception rate rate was 92.3%, whereas the infertility rate was observed at 7.7%. The average fecundity rate in the AKK lambs was 1.04, whereas it was 1.16 for the CAM lambs. Fecundity at weaning peaked in 2017 for the AKK lambs at 1.05 and in 2015 for the CAM lambs at 1.14. The average fecundity at weaning for both breeds was calculated at 0.98 and 1.04, respectively. On average, the litter size for both breeds was calculated at 1.12 and 1.26 for AKK and CAM, respectively. Similarly, the litter size at weaning peaked in 2017 for the AKK lambs at 1.10 and in 2015 for the CAM lambs at 1.17. The average litter size at weaning for both breeds was determined to be 1.05 and 1.12 for AKK and CAM, respectively.

## 4 Discussion

### 4.1 Breed Effect on Growth Traits

As seen in Table 2, BW of the AKK breed was higher compared to the CAM breed. According to various studies, BWs in the AKK breed have been reported to range between 3.87 and 4.58 kg (Aksoy et al. 2023; Noyan and Ceyhan 2021; Sakar and Ünal 2021). Literature studies on crossbreeds obtained through similar crossbreeding practices as with the CAM breed, resulting in Merino‐derived hybrids, reported BWs ranging from 3.10 to 4.58 kg (Aktaş et al. 2015, 2016; Bülbül et al. 2014; Dixit, Dhillon, and Singh 2001; Mallick et al. 2017; Muller et al. 2023). Additionally, for the Merino breed, live weight was reported as 3.98 kg, which is lower than the result in the current study (Cloete et al. 2021). Overall, the BWs of both breeds seem consistent with findings from other researchers. Lamb BW is a crucial factor influencing the postnatal survival and growth potential of lambs (Hinch et al. 1985). The higher BW of the AKK breed compared to the CAM breed can also be explained by differences in the ewe's capacity to use nutrition during the later stages of pregnancy within the CAM breed.

As depicted in Table 2, the AKK lambs exhibited higher BWs compared to the CAM lambs but displayed lower values for WW, ADWG and the KR. Studies on 90‐day WWs in Black Face Merinos, CAM and Malya lambs reported weights of 30.29, 31.83 and 28.68 kg, respectively, which were higher than the recorded WWs of both the AKK and CAM breeds in this study (Ceyhan et al. 2009).

In studies, including Bharat Merinos, Malpura and AKK lambs, WWs ranged between 13 and 24 kg, which are lower than the values reported on in this study (Ceyhan et al. 2019; Dixit, Dhillon, and Singh 2001; Noyan and Ceyhan 2021; Chopra et al. 2010). Similarly, the WWs of AKK and AKK × Sakız crossbred lambs (26.38 and 25.48 kg) were lower compared to this study (Ünal 2002). However, the present study's WWs for both breeds align closely with reported values in Karacabey Merino lambs (Sezenler et al. 2013). These findings, along with those of abovementioned researchers, suggest that differences in WWs might be attributed to various environmental conditions reflected in rearing practices, ranging from geographical variations to pasture characteristics. Additionally, genotypes, nutrition, herd management and breeding strategies could contribute to these variations, elucidating the differences observed in WWs.

The ADWG of the CAM breed was approximately 11 g higher than that of the AKK breed (Table 2). In studies focusing on meat sheep breeds, such as the Danish Texel, Shropshire, Oxford Down and Suffolk breeds (Maxa et al. 2007), the observed ADWG ranged from 281 to 333 g, surpassing the results reported on in this study. In contrast, other studies indicated lower ADWG values for breeds like Awassi, Awassi × Romanov (0.191 and 0.152 kg), AKK (0.222 kg) and CAM (191.0 g) (Aktaş et al. 2016; Noyan and Ceyhan 2021; Türkyılmaz et al. 2021).

The KR, a trait often used to assess feed efficiency, serves as an indicative measure of growth performance, especially WW and ADWG. In Table 2, it is evident that the KR value was higher for the CAM breed compared to the AKK breed. The KR values of both breeds were found to exceed those reported on in other studies (Eskandarinasab, Ghafouri‐Kesbi, and Abbasi 2010; Mahala et al. 2020).

### 4.2 Fixed Effects

The study revealed a significant sex effect on the BW, WW, ADWG and KR traits (*p* < 0.001), with males consistently exhibiting higher values than females across all the traits. This result aligns with findings from various studies on sheep breeds (Sezenler et al. 2013; Aktaş et al. 2015; Noyan and Ceyhan 2021; Muller et al. 2023; Eskandarinasab, Ghafouri‐Kesbi, and Abbasi 2010) that also observed higher BW, WW, ADWG and KR values in male lambs compared to female lambs. The elevated BW in males may be attributed not only to genotype but also to potential influences stemming from the additional care and nutrition provided during the pregnancy period to ewes (Koyuncu and Duymaz 2017). Additionally, the higher WW and ADWG values in males can be attributed to the more effective endocrine system, particularly regarding the roles of testosterone and oestrogen. Testosterone facilitates greater growth in males, whereas oestrogen in females may limit bone growth (Assan 2020). Furthermore, WW and ADWG indirectly impact the KR characteristic.

The study revealed significant effects of birth type on BW and WW (*p* < 0.001), with the KR value also showing significance (*p* < 0.05). However, ADWG was not significant (Table 2). Previous studies on AKK lambs (Aktaş and Doǧan 2014; Noyan and Ceyhan 2021) and Karacabey Merino lambs (Sezenler et al. 2013) have similarly reported on the significant influence of birth type on BW and WW (*p* < 0.001). Correspondingly, research on Bharat Merino lambs (Chopra et al. 2010) contributes to these findings, emphasizing the significant impact of birth type on BW and WW while regarding ADWG as non‐significant. Conversely, studies by Sakar and Ünal (2021) on AKK lambs and by Yilmaz and Akmaz (2000) on Konya Merino lambs indicated the statistical insignificance of birth type, particularly at 90 days.

Regarding KR, akin to our study, research by Eskandarinasab, Ghafouri‐Kesbi, and Abbasi (2010) and Supakorn and Pralomkarn (2012) highlighted the significance of birth type. The necessity for supplemental feeding for milk production in pregnant ewes delivering multiplets during gestation and postpartum periods was acknowledged (Vatankhah and Talebi 2009). However, the practical implementation of this practice in breeding conditions poses challenges, potentially resulting in growth disparities between twin‐born lambs and their single‐born counterparts. Moreover, variations in flock management practices among farms are believed to contribute to this observed impact.

The statistical analysis revealed a significant influence of dam age across all measured traits (*p* < 0.001). Although certain investigations on Karacabey Merino and Kıvırcık lambs reported an inconsequential impact of dam age on BW, notably, its significance emerged in shaping lambs’ characteristics at 90 days of age (Koyuncu and Uzun 2009). In contrast, studies on Alpine Merino, CAM and Chinese Superfine Merino lambs (Aktaş et al. 2015; Di et al. 2011; Li et al. 2022) underscored the substantive role of maternal age across all traits, aligning with the current study's findings. Conversely, research on German Blackhead Meat × Karacabey Merino and Lori‐Bakhtiari lambs (Ceyhan et al. (2009); Vatankhah and Talebi 2009) reported on the non‐significant impact of dam age. Consistent evidence from multiple studies supports the assertion that dam age significantly influences pre‐weaning growth characteristics. Moreover, factors such as the dam's physical condition and live weight contribute meaningfully to lamb growth attributes (Chopra et al. 2010). It is suggested that an amalgamation of factors affecting milk production, including dam age, optimal physical condition during parturition, maternal expertise, managing twin or single lambs and genotypic disparities, collectively govern pre‐weaning growth characteristics.

The observed impact of birth year on growth traits aligns with the findings from various studies across both similar and dissimilar breeds, including the AKK (Noyan and Ceyhan 2021; Öztürk et al. 2018), Karayaka (Tamer and Şirin 2021), Afshari (Eskandarinasab, Ghafouri‐Kesbi, and Abbasi 2010), CAM (Aktaş et al. 2016), Awassi (Gül et al. 2020), Dorper × indigenous sheep (Teklebrhan et al. 2014) and Karacabey Merino (Sezenler et al. 2013) breeds. Notably, statistically significant trends have been consistently observed across these studies. The variations noted in pasture areas due to dynamic climatic conditions over successive years, alongside the fluctuations in flock management practices and feeding regimes, are posited to have contributed significantly to the outcomes observed in this investigation.

The effect of herd size across all measured traits (*p* < 0.001) highlights an intriguing trend. The larger scale sheep farming operations consistently demonstrate a superior performance across the traits delineated in this study (Table 2). This observation aligns with the economic principle suggesting that as sheep farming enterprises expand, there is a concomitant decrease in unit costs alongside an upsurge in sales revenue and overall profitability (Tamer and Sariözkan 2017). Consequently, this study corroborates previous findings, indicating that the enhanced economic viability of larger enterprises corresponds to a more pronounced emphasis on lamb care and feeding practices compared to their counterparts operating on smaller scales. Moreover, larger operations tend to implement herd management practices more effectively.

Regarding the impact of birthing seasons on growth traits, the statistically significant difference observed in growth traits between lambs born in winter (December, January and February) and those born in spring (March, April and May) underscores the substantial influence of birthing seasons on these traits (*p* < 0.001). In comparative studies, Thiruvenkadan et al. (2011) reported similar BW findings in Mecheri Sheep to this study but noted an insignificant effect of birth season on WW. Lakew, Melekot, and Mekuriaw (2010) observed an insignificant impact on birth weight (BW) for local and Dorper × local crossbred sheep but noted a non‐significant effect on WW. Findings on Awassi (Gül et al. 2020) and Dorper × indigenous sheep breeds (Teklebrhan et al. 2014) were similar to this study, indicating a substantial effect of seasonality on both BW and WW. During winter, sheep farms commonly adopt enclosed barn practices due to harsh conditions, whereas in spring, allowing sheep and lambs greater access to grazing areas reduces stress by avoiding damp conditions. The observed impact of the spring season on lamb growth attributes underscores the favourable conditions provided within barns during this period.

### 4.3 Lamb Livability

In the current study, the survival rates of lambs from birth to weaning were significantly influenced by breed, birth type and birth year. Specifically, AKK lambs demonstrated a higher survival rate (93.85%) compared to CAM lambs (89.50%), which aligns with findings in previous studies that suggest certain breeds possess innate resilience against environmental and health‐related challenges (Smith and Jones 2018; Taylor et al. 2020). The higher survival rate in AKK lambs could be attributed to their adaptive traits to the local environmental conditions in Türkiye, as suggested by Ozturk et al. (2019).

The survival advantage of single‐born lambs over twins (95.46% vs. 82.11%) supports the well‐documented phenomenon where single‐born lambs typically receive more maternal care and nutrition, leading to better survival outcomes (Brown et al. 2017). This is consistent with the findings of Thompson et al. (2016), who reported that single‐born lambs have a lower risk of mortality due to reduced competition for resources.

Although our analysis revealed statistically significant variations in survival rates across different years, factors, such as gender, dam age, herd size and birth season, did not significantly affect lamb survival rates (*p* > 0.05). This contrasts with some literature suggesting that these factors can influence lamb mortality under different management and environmental conditions (Walker and Sawyer 2021). The lack of significant effects in our study might be due to the relatively uniform management practices and environmental conditions across the herds examined.

These findings highlight the importance of breed selection and managing birth types to enhance lamb survival rates. Further research could explore the specific genetic and environmental interactions that contribute to these differences, providing more insights into optimizing management practices for different breeds (Kumar et al. 2021).

### 4.4 Reproductive Performance

The conception rates between the AKK and CAM breeds observed in this study are consistent with the broader range reported in the literature, indicating close proximity. In comparison, previous studies have reported conception rates of 86.6% for the CAM breed (Aktaş et al. 2015) and 90.1% for the AKK breed (Ceyhan et al. 2019). Other breeds, such as Karayaka (86.2%) (Tamer and Şirin 2021), Awassi (83.3%) and Romanov/Awassi crossbred (91.9%) (Türkyılmaz et al. 2021), also show varying conception rates. Interestingly, the reported conception rates for the CAM breed (86.9%) (Kırbaş, Bülbül, and Kal 2022) and AKK breed (83.67%) (Ünal 2002) are lower than those observed in this study. Similarly, the AKK breed has previously reported conception rates of 92.6% (Oğrak 2020) and 90.2% (Aksoy et al. 2023), aligning closely with our findings.

The variation in conception rates across studies may be attributed to factors, such as management practices, genetic differences and environmental conditions. Effective monitoring of non‐pregnant individuals and optimal nutrition during mating seasons are crucial strategies that can enhance conception rates, as suggested by the current findings.

Regarding fecundity and fecundity at weaning, the CAM breed exhibited notably higher values than the AKK breed in this study. The fecundity of the AKK breed (1.01) (Ceyhan et al. 2019) and the fecundity (1.03) and fecundity at weaning (0.96) (Aksoy et al. 2023) are consistent with our results. Similarly, the CAM breed's fecundity (1.12) and fecundity at weaning (1.03) (Kırbaş, Bülbül, and Kal 2022) corroborate our findings.

Furthermore, the CAM breed showed a higher litter size and litter size at weaning compared to the AKK breed (Table 3). Although Tüfekci (2023) reported a higher litter size for the AKK breed (1.22) than observed in this study, it was still lower than that of the CAM breed. Aksoy et al. (2023) also reported similar findings for AKK breed litter size (1.13) and litter size at weaning (1.05). In contrast, Kırbaş, Bülbül, and Kal (2022) documented higher litter sizes for the CAM breed (1.30) and litter size at weaning (1.19) than those observed in this study.

These variations in litter size and litter size at weaning can be influenced by several factors, including feeding practices, pasture conditions and genetic makeup. Enhanced feeding strategies and proper pasture management have been shown to improve reproductive performance and lamb growth (Smith and Jones 2018). Further research could explore the genetic and environmental interactions contributing to these differences, providing insights for optimizing breeding and management practices in different sheep breeds.

## 5 Conclusion

In conclusion, this study demonstrated that crossbreeding AKK with German Meat Merino has effectively combined the adaptability traits of AKK with the enhanced meat and wool production qualities of the German Meat Merino, resulting in the CAM breed. Our comprehensive comparison of pre‐weaning growth traits (BW, WW and ADWG) and the KR indicated that although the AKK breed had a higher birth weight (BW), the CAM breed excelled in WW, ADWG and KR. Additionally, conception rate and survival rates were higher in the AKK breed, whereas the CAM breed showed superior performance in reproductive parameters, such as fecundity, fecundity at weaning, litter size and litter size at weaning. The significant influence of fixed effects on pre‐weaning growth traits and KR, with the exception of birth type on ADWG, underscores the importance of environmental and management factors in shaping these traits. The alignment of our findings with studies on similar breeds and the differences observed with studies on other breeds highlight the impact of genotype–environment interactions. Key environmental factors, including annual climatic variations, pasture conditions and market dynamics, play crucial roles in determining the growth and reproductive characteristics of these breeds. To sustain and enhance the success of breeding programmes, a thorough understanding of genotype–environment interactions is essential. The meticulous maintenance of pedigree records is critical for calculating specific trait heritabilities, aiding in precise selection and breeding decisions. Furthermore, the growing interest in global genomic selection emphasizes the need for comprehensive record‐keeping and the establishment of reference populations for each breed. As small ruminant farming remains vital to food security and rural economies, ongoing selection and breeding programmes are crucial, especially given the challenges posed by an increasing global population.

## Author Contributions

Material preparation, data collection, analysis and writing the manuscript were performed by Sedat Behrem.

## Ethics Statement

According to the Regulation on the Working Procedures and Principles of Animal Experiments Ethics Committees dated 15.02.2014 and numbered 28914 of the Ministry of Forestry and Water Affairs, since this study does not include any clinical practice or patient data, there is no need for an ethics committee decision.

## Conflicts of Interest

The authors declare no conflicts of interest.

## Data Availability

The data that support the findings of this study are available from the corresponding author upon reasonable request.
